# Quantitative estimation of pulmonary artery wedge pressure from chest radiographs by a regression convolutional neural network

**DOI:** 10.1007/s00380-022-02043-w

**Published:** 2022-02-27

**Authors:** Yuki Saito, Yuto Omae, Daisuke Fukamachi, Koichi Nagashima, Saki Mizobuchi, Yohei Kakimoto, Jun Toyotani, Yasuo Okumura

**Affiliations:** 1 Division of Cardiology, Department of Medicine, Nihon University School of Medicine, 30-1 Ohyaguchi-kamicho, Itabashi-ku, Tokyo, 173-8610 Japan; 2 Department of Industrial Engineering and Management, College of Industrial Technology, Nihon University, Chiba, Japan

**Keywords:** Artificial intelligence, Deep learning, Heart failure, Diagnostic method

## Abstract

Recent studies reported that a convolutional neural network (CNN; a deep learning model) can detect elevated pulmonary artery wedge pressure (PAWP) from chest radiographs, the diagnostic images most commonly used for assessing pulmonary congestion in heart failure. However, no method has been published for quantitatively estimating PAWP from such radiographs. We hypothesized that a regression CNN, an alternative type of deep learning, could be a useful tool for quantitatively estimating PAWP in cardiovascular diseases. We retrospectively enrolled 936 patients with cardiovascular diseases who had undergone right heart catheterization (RHC) and chest radiography and estimated PAWP by constructing a regression CNN based on the VGG16 model. We randomly categorized 80% of the data as training data (training group, *n* = 748) and 20% as test data (test group, *n* = 188). Moreover, we tuned the learning rate—one of the model parameters—by 5-hold cross-validation of the training group. Correlations between PAWP measured by RHC [ground truth (GT) PAWP] and PAWP derived from the regression CNN (estimated PAWP) were tested. To visualize how the regression CNN assessed the images, we created a regression activation map (RAM), a visualization technique for regression CNN. Estimated PAWP correlated significantly with GT PAWP in both the training (*r* = 0.76, *P* < 0.001) and test group (*r* = 0.62, *P* < 0.001). Bland–Altman plots found a mean (SEM) difference between GT and estimated PAWP of − 0.23 (0.16) mm Hg in the training and − 0.05 (0.41) mm Hg in the test group. The RAM showed that our regression CNN model estimated high PAWP by focusing on the cardiomegaly and pulmonary congestion. In the test group, the area under the curve (AUC) for detecting elevated PAWP (≥ 18 mm Hg) produced by the regression CNN model was similar to the AUC of an experienced cardiologist (0.86 vs 0.83, respectively; *P* = 0.24). This proof-of-concept study shows that regression CNN can quantitatively estimate PAWP from standard chest radiographs in cardiovascular diseases.

**Supplementary Information:**

The online version contains supplementary material available at 10.1007/s00380-022-02043-w.

## Introduction

The prevalence of heart failure is increasing, and high rates of mortality and hospital admissions due to heart failure represent a major burden on health care systems [1]. In heart failure, elevated left atrial pressure causes pulmonary congestion, and pulmonary artery wedge pressure (PAWP) measured by right heart catheterization (RHC) is widely used as a surrogate for left atrial pressure and was found to be directly associated with severity and prognosis of heart failure [2, 3]. However, RHC is an invasive procedure with a potential risk for complications; therefore, a noninvasive method is needed to assess PAWP.

Chest radiography is the most common diagnostic imaging tool in medicine and has been used as the first-line test for detecting elevated PAWP [4]. Abnormal signs on chest radiographs, such as increased cardiothoracic ratio, alveolar and interstitial edema, and dilated left atrium, were reported to be associated with elevated PAWP [4]. However, the interpretation of chest radiographs is subjective and depends on the experience of the physician; often, general physicians have difficulty assessing PAWP by chest radiographs [5, 6].

With the recent development of artificial intelligence (AI), deep learning has become a powerful tool to assist with diagnosis in medicine. Convolutional neural network (CNN) is a traditional type of deep learning model for processing data that have a grid pattern, such as images, and is designed to automatically extract features from low- to high-level patterns [7]. CNN has become an effective method for detecting and classifying various diseases [8–10]. In the field of cardiovascular medicine, recent studies found that CNN was useful for detecting cardiomegaly, heart failure, and elevated PAWP from chest radiographs [11–13]. However, it is not able to quantitatively estimate PAWP.

Recently, regression CNN, an alternative type of deep learning, has been used in the field of radiology to quantitatively estimate age from radiographs [14]. Regression CNN is a method for training a network to perform linear regression on data rather than simply classifying them. In the present study, we hypothesized that a regression CNN that uses chest radiographs could be useful for quantitatively estimating PAWP in patients with cardiovascular diseases. Therefore, we performed a study with the aim to create, train, and test a novel regression CNN method for estimating PAWP from standard chest radiographs.

## Materials and methods

### Study participants

We retrospectively enrolled 936 patients with cardiovascular diseases who had undergone both RHC and chest radiography between January 2017 and December 2019 at Nihon University Itabashi Hospital, Tokyo, Japan. RHC was performed to accurately monitor hemodynamic status, and treatment was not changed between the two procedures. The exclusion criteria included respiratory diseases (lung tumor, postpneumonectomy, pneumonia, and tuberculosis), percutaneous cardiopulmonary support, a left ventricular assist device, and unstable clinical conditions that required emergency cardiac catheterization. Paired RHC data and chest radiograph images were collected.

### Data collection

RHC was performed with a Swan-Ganz catheter in either the cardiac catheterization laboratory or the cardiac care unit. Pressure calibration was performed, and pressure was measured at end-expiration while the patient was supine. The following hemodynamic parameters were measured: PAWP, pulmonary artery systolic pressure, mean pulmonary artery pressure, cardiac index, and cardiac output (cardiac output was measured by thermodilution or the Fick method).

**Fig. 1:**
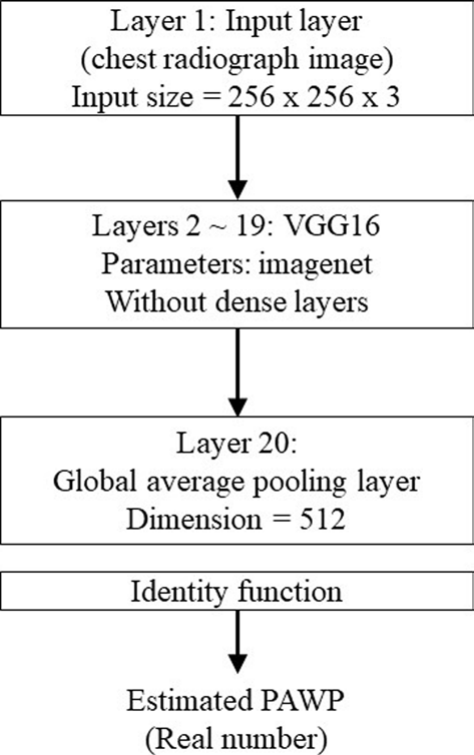
Regression convolutional neural network. The structure of the regression convolutional neural network used to estimate pulmonary artery wedge pressure

Chest radiography was performed in a standing or sitting position. A Digital Imaging and Communications in Medicine (DICOM) image of the chest radiograph was available for each patient. All DICOM images were transformed to 256 × 256 pixels. We then used a regression CNN to estimate PAWP as a real number (see Fig. 1). The VGG16 model is one of the popular models for transfer learning, so we used it with the ImageNet parameters as layers 2–19 of the model (we did not use the flattened and dense layers of the VGG16) [15]. VGG16 is a classification system based on 3-channel color images. The size of layer 1 (the input layer) is 256 × 256 × 3 pixels. Because the chest radiograph image is a 1-channel, grayscale image, we stacked three identical images on top of each other to create a color image. After analysis by VGG16, the convoluted images were transitioned to a global average pooling (GAP) layer, which is layer 20. Using a GAP layer, we were able to create a regression activation map (RAM) to visualize in the form of a heatmap how our deep learning model estimated PAWP [16]. Because the aim of the study was to develop a regression CNN for estimating PAWP as a real number, we set the identity function as the activation function of the output layer. Creating a RAM is a recently developed method to produce a heatmap that highlights the regions in the image where the regression CNN model focuses.

### Preparation of training and test data sets

**Fig. 2:**
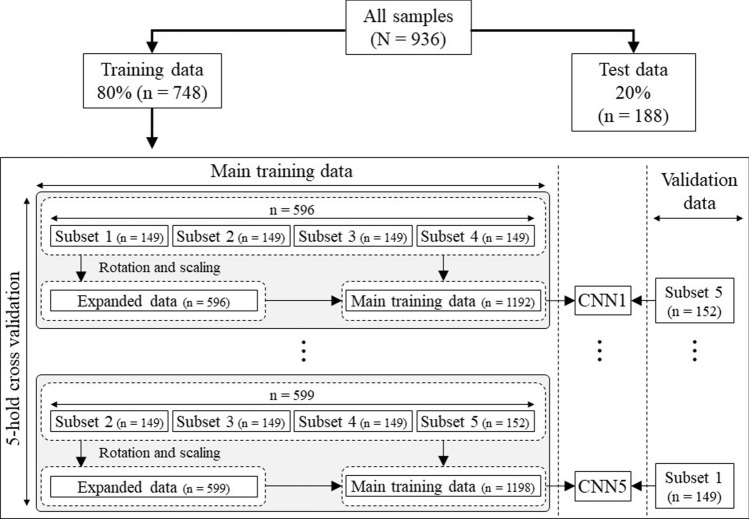
Study flowchart. To develop a regression convolutional neural network and verify its generalization error, we randomly categorized all data (*N* = 936) as training data and test data; 80% of all data were categorized as training data (training group, *n* = 748) and 20% as test data (test group, *n* = 188). The training data were split into 5 subsets for 5-hold cross-validation

To develop a regression CNN and estimate its generalization error, we randomly categorized all data (*N* = 936) as training data or test data (Fig. 2). In this process, we developed a CNN using four subsets (the main training data) and validated it with a rest subset (the validation data). To achieve high accuracy, we created expanded data by randomly rotating images by plus and minus 10 degrees and randomly scaling the images by plus and minus 5%. The size of the expanded data and the original images was the same. For example, if subset 5 was to be validated, the total number of images in subsets 1–4 was 596 images, and the expanded data also included 596 images. Therefore, the total number of images in the main training data was 1192 images (596 original images and 596 expanded data images). The above procedures were performed for cross-validation. The learning condition was set as minibatch learning (batch size = 8), root mean square propagation (RMSprop) was performed for optimization, and mean squared error (MSE) was set as the cost function to measure performance of the model; 200 epochs were run.

### Construction of the regression CNN

The learning rates of RMSprop were set as 10^–5.5^, 10^–6.0^, and 10^–6.5^; Supplementary Fig. 1 shows the learning results of these three learning rates; each graph has five lines because we performed fivefold cross-validation. The cross-validation MSEs of the validation data for 200 epochs of the learning rates 10^–5.5^, 10^–6.0^, and 10^–6.5^ were 31.99, 33.53, and 31.71, respectively. Therefore, we considered a learning rate of 10^–6.5^ to be optimal. Furthermore, to avoid overfitting, we searched in the range from 1 to 200 epochs for the number of epochs that resulted in the minimum cross-validation MSE and found that 150 was the optimal number. With 150 epochs, the minimum cross-validation MSE was 30.25. Thus, we adopted the learning rate 10^–6.5^ and 150 epochs for our regression CNN.

Deep learning was performed with Python version 3.8.9 (Python Software Foundation, Beaverton, OR, USA) and Keras version 2.7.0 software (GitHub, San Francisco, CA, USA). As the backend of Keras, we used TensorFlow 2.7.0, a deep learning framework. We built the regression CNN on a Linux computer (operating system, Ubuntu 20.04; central processing unit, Intel Core i7-10750H; random access memory, 16 GB; graphics processing unit, NVIDIA GeForce RTX 3060).

### Statistical analysis

Continuous variables were expressed as medians [interquartile range (IQR)] and compared by a Mann–Whitney test. Categorical variables were expressed as number and percentage of patients and compared by a chi-squared test. Correlations between PAWP measured by RHC [ground truth (GT) PAWP] and PAWP estimated by the regression CNN (estimated PAWP) were tested by Pearson’s correlation coefficient. Bland–Altman plots were used to show agreement between GT and estimated PAWP by plotting the difference against the mean. Receiver-operating characteristic (ROC) curves were generated, and the area under the curves (AUC) was determined as a measurement of the ability of the regression CNN model to detect elevated PAWP. We defined elevated PAWP as a value greater than or equal to 18 mmHg [17]. The diagnostic ability of the regression CNN model and an experienced cardiologist, who was blind to the PAWP data, were evaluated by AUCs, and the AUCs were compared by the Delong method [18]. All statistical analyses were performed with JMP 13.0 (SAS Institute, Cary, NC, USA) and the MedCalc Software program, Version 18.5 (MedCalc Software, Mariakerke, Belgium). For all analyses, *P* < 0.05 was considered statistically significant.

## Results

### Patient characteristics

The clinical diagnoses and indication for RHC of the 936 patients are presented in Table 1. In our cohort, 593 (63.3%) patients had ischemic heart disease and 193 (20.6%) patients had heart failure. The clinical characteristics of the training and test groups are shown in Table 2. We found no significant difference between the two groups in age, sex, body mass index, body surface area, or heart rate. Hemodynamic parameters, including systolic blood pressure, cardiac output, cardiac index, and PAWP, were also not significantly different between the two groups. The distribution of PAWP among the study patients is shown in Supplementary Fig. 2. The median PAWP value was 11 mm Hg (IQR 7–15 mm Hg) in the training group and 11 mm Hg (IQR 8–14 mm Hg) in the test group (*P* = 0.84).

**Table 1:** Clinical diagnosis and indication for right-sided cardiac catheterization

Clinical diagnosis	*N* = 936
Ischemic heart disease, *n* (%)	593 (63.3)
Heart failure, *n* (%)	193 (20.6)
Valvular heart disease, *n* (%)	108 (11.5)
Hypertrophic obstructive cardiomyopathy, *n* (%)	12 (1.2)
Pulmonary arterial hypertension, *n* (%)	7 (0.7)
Arrhythmia, *n* (%)	4 (0.4)
Atrial septal defect, *n* (%)	5 (0.5)
Others, *n* (%)	14 (1.5)

**Table 2:** Clinical characteristics of patients

Item	Training group (*n* = 748)	Test group (*n* = 188)	*P* value
Age, median (IQR), y	71 (62–77)	71 (60–78)	0.67
Male, *n* (%)	582 (77.8)	140 (74.7)	0.33
Body mass index, median (IQR), kg/m^2^	23.4 (20.8–26.0)	23.6 (21.4–26.0)	0.26
Body surface area, median (IQR), m^2^	1.71 (1.56–1.85)	1.71 (1.58–1.90)	0.50
Heart rate, median (IQR), bpm	69 (62–78)	68 (60–78)	0.46
Systolic blood pressure, median (IQR), mmHg	126 (109–143)	127 (111–145)	0.61
CO, median (IQR), L/min	4.4 (3.7–5.4)	4.6 (3.8–5.5)	0.55
CI, median (IQR), L/min/m^2^	2.6 (2.2–3.1)	2.6 (2.2–3.1)	0.35
PAWP, median (IQR), mmHg	11 (7–15)	11 (8–14)	0.84

*CI* cardiac index, *CO* cardiac output, *IQR* interquartile range, *PAWP* pulmonary arterial wedge pressure

### Validation of the regression CNN

**Fig. 3:**
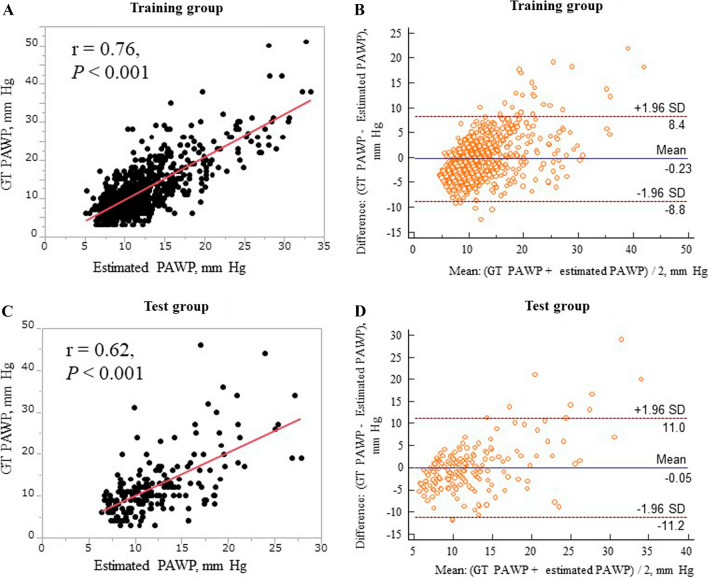
Relation between ground truth and estimated pulmonary artery wedge pressure in the training and test groups. **A** Scatter plots showing the relation between ground truth (GT) and estimated pulmonary artery wedge pressure (PAWP) in the training group. **B** Bland–Altman plot of the training group data. **C** Scatter plots showing the relation between GT and estimated PAWP in the test group. **D** Bland–Altman plot of the test group data. *GT* ground truth, *PAWP* pulmonary artery wedge pressure

In the training group, estimated PAWP derived from the regression CNN significantly correlated with GT PAWP (*r* = 0.76, *P* < 0.001, Fig. 3A). Bland–Altman plots found a mean (SEM) difference between the GT and estimated PAWP of − 0.23 (0.16) mm Hg, the upper limit of agreement [defined as mean (+ 1.96 SD)] was 8.4 mm Hg, and the lower limit of agreement [defined as mean (− 1.96 SD)] was − 8.8 mm Hg (Fig. 3B). However, when the mean GT PAWP and estimated PAWP were over 30 mm Hg, which was the case in several patients, the estimated PAWP tended to be underestimated.

In the test group, estimated PAWP derived from the regression CNN significantly correlated with the GT PAWP (*r* = 0.62, *P* < 0.001, Fig. 3C). Bland–Altman plots found a mean (SEM) difference between GT and estimated PAWP of − 0.05 (0.41) mm Hg, the upper limit of agreement [defined as mean (+ 1.96 SD)] was 11.0 mm Hg, and the lower limit of agreement [defined as mean (− 1.96 SD)] was − 11.2 mm Hg (Fig. 3D). As in the training group, several patients in the test group had a mean GT and estimated PAWP over 30 mm Hg, and the estimated PAWP in these patients tended to be underestimated.

### Assessment of the RAM

**Fig. 4:**
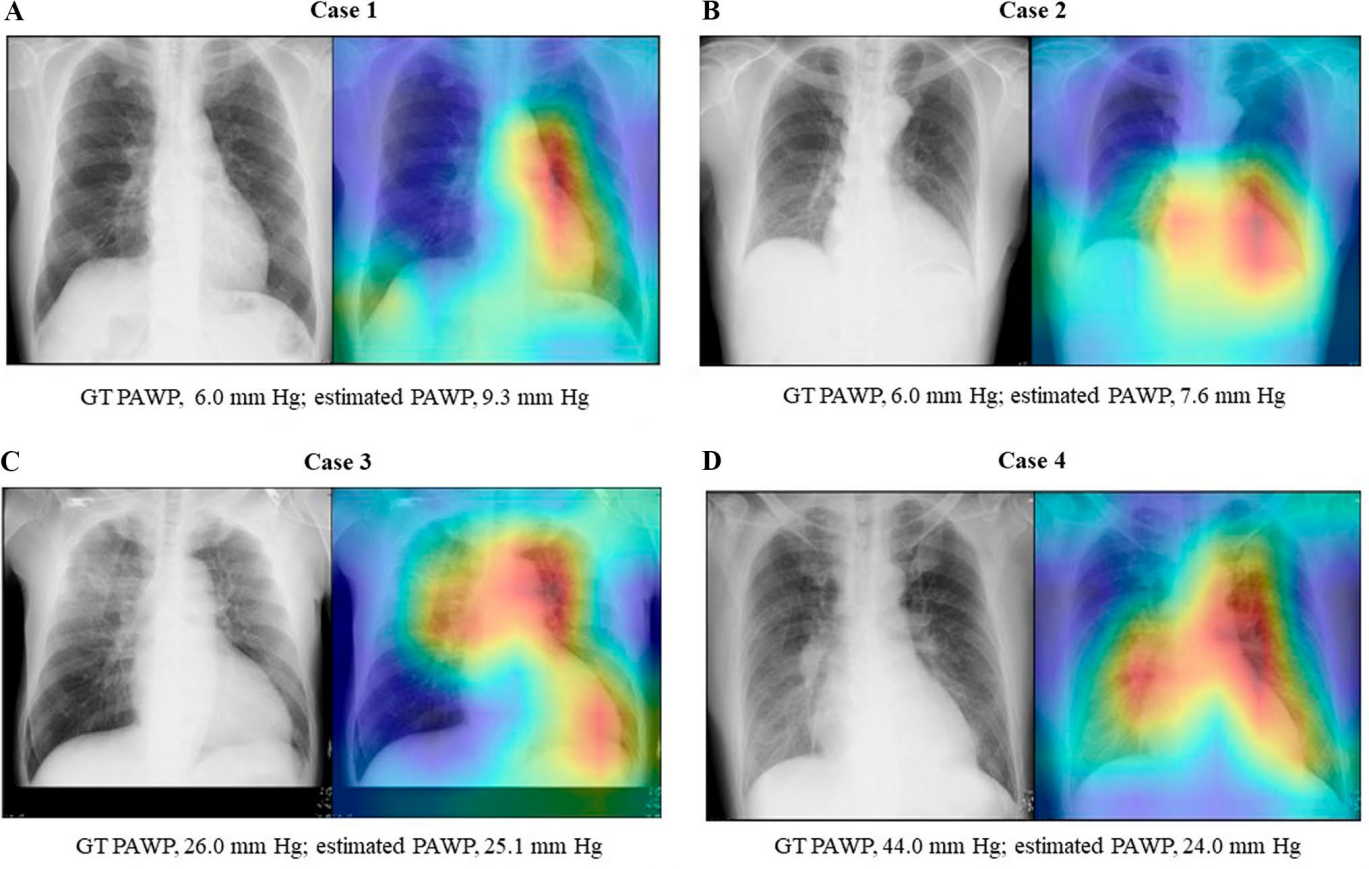
Representative cases. Examples of visualization with a regression activation map (RAM). In each case, the original image is on the left and its heatmap is on the right. The red and yellow areas on the heatmap represent the points on which the regression CNN model focused. **A** Case 1: A 73-year-old man with ischemic heart disease. Ground truth (GT) pulmonary artery wedge pressure (PAWP), 6.0 mm Hg; estimated PAWP, 9.3 mm Hg. **B** Case 2: A 69-year-old man with ischemic heart disease. GT PAWP, 6.0 mm Hg; estimated PAWP, 7.6 mm Hg. **C** Case 3: A 60-year-old man with ischemic heart disease. GT PAWP, 26.0 mm Hg; estimated PAWP, 25.1 mm Hg. **D** Case 4: A 55-year-old man with heart failure. GT PAWP, 44.0 mm Hg; estimated PAWP, 24.0 mm Hg. *GT* ground truth, *PAWP* pulmonary artery wedge pressure

To visualize how the regression CNN assessed the images, we used an RAM to analyze where AI focused in the images. Representative cases in the test group are shown in Fig. 4. The red and yellow areas on the heatmaps show the regions where AI focused to determine the estimated PAWP. In most cases, our regression CNN model focused only on the left side of the cardiac area; however, in several cases with high GT PAWP (e.g., cases 3 and 4 in Fig. 4), our regression CNN model focused not only on the cardiac area but also on the pulmonary congestion.

### Detection of elevated PAWP by the regression CNN model and the cardiologist

When we compared the AUCs for detecting elevated PAWP produced by the regression CNN model and the cardiologist in test group, we found that the AUC produced by the regression CNN model was similar to that of the cardiologist (0.86 vs 0.83, respectively; *P* = 0.24).

## Discussion

This is the first clinical study to propose a method for quantitatively estimating PAWP using a regression CNN with standard chest radiographs in patients with cardiovascular diseases. Our study has three main findings. First, regression CNN can calculate estimated PAWP as a numerical value from standard chest radiographs; second, estimated PAWP moderately correlates with GT PAWP obtained by RHC; and third, as visualized by an RAM, regression CNN focuses on cardiomegaly and pulmonary congestion to estimate PAWP, and the diagnostic performance of the regression CNN is similar to that of an experienced cardiologist. This proof-of-concept study showed that regression CNN can potentially be used to quantitatively estimate PAWP from standard chest radiographs.

PAWP has been reported to be one of the most important hemodynamic parameters in heart failure [19, 20]. Traditionally, physicians have subjectively estimated PAWP by estimating the severity of pulmonary edema from chest radiographs (the standard noninvasive screening method for assessing pulmonary congestion) [4]. However, besides being subjective, this approach is unreliable because standard chest radiography has low sensitivity for estimating the severity of pulmonary congestion [5, 21]. A recent clinical study reported that the redistribution of pulmonary perfusion associated with pulmonary congestion was seen in a ventilation/perfusion single-photon emission computed tomography even in cases where a thoracic radiology specialist could not detect pulmonary congestion [6]. Consequently, standard chest radiography has limited value for objectively estimating PAWP.

With the development of artificial intelligence and deep learning in recent years, interest has grown in using these technologies in clinical research. The most established, traditionally used algorithm among the various deep learning models is CNN [7]. CNN is a class of artificial neural networks that is typically composed of three types of layers, i.e., convolution, pooling, and fully connected layers. Feature extraction is performed in the convolution and pooling layers, and output, such as classification of images, is performed in the fully connected layer [7]. CNN has become an effective method for classification tasks and was recently applied for diagnosing images in various clinical fields in medicine. For example, CNN has been used to classify skin diseases from skin photographs and optic disc abnormalities from fundus photographs [22, 23].

Several recent studies have reported on using CNN for diagnosing cardiovascular diseases. One recent, interesting clinical study showed the usefulness of a CNN model for diagnosing heart failure from chest radiographs [12]. In addition, another recent study reported on the use of a CNN algorithm with chest radiographs to identify elevated PAWP [13]. Although these CNN models provide qualitative assessments of PAWP, they are unable to quantitatively estimate PAWP from chest radiographs.

Regression CNN is an alternative deep learning method that can output a real number from digital image data such as radiographs. It uses convolutional layers to convert the inputted image data into small-scale data, which are flattened by the middle, fully connected layers and ultimately transformed into a real number. This approach is used in the field of radiology, for example to quantitatively estimate pediatric bone age from radiographs [24]. Another example is the use of regression CNN to predict a patient’s age from chest radiographs in emergency medicine [14]. In the present study, we applied this method to quantitatively estimate PAWP from chest radiographs in patients with cardiovascular diseases. Our findings showed the potential usefulness of this approach because the PAWP estimated by the model from chest radiograph image data correlated with the actual PAWP measured by RHC.

A quantitative approach is more sensitive and objective than a qualitative approach, so it may be more useful for decision making in heart failure management. PAWP is commonly also assessed by echocardiography; however, this method has relevant shortcomings, including dependence on the examiner’s skill, time, and cost. Furthermore, even a detailed study of an echocardiogram by an expert sonographer in accordance with the current guidelines provides only a semiquantitative assessment [25, 26].

To enable the safe application of deep learning in medical imaging, it is important to identify and visualize how the deep learning process assesses and learns from images. To visualize the features on which deep learning focuses, several techniques are available, such as gradient-class activation maps, Generative Visual Rationals, and RAM [27, 28]. In the present study, we used RAM, a state-of-the-art method that provides a visual explanation of how regression CNN has learned the features of images [16]. Our results revealed that our regression CNN assessed PAWP by focusing on the left side of the cardiac region and on the pulmonary congestion. This assessment approach was considered to be similar to that of a cardiologist. To our knowledge, this study is the first to apply RAM to medical imaging in the field of cardiology.

Our results suggested that a regression CNN model can estimate PAWP on the basis of only one DICOM image of a chest radiograph. This mehod is noninvasive, fast, simple, objective, and inexpensive and has the potential to be clinically useful by assisting physicians in various clinical situations, including screening for heart disease during medical check-ups, managing heart failure, and treating outpatients. Applying regression CNN to chest radiography will make it a more useful and beneficial diagnostic tool in medicine.

The present study has several limitations. First, it was a single-center study with a relatively small sample size. Generally, deep learning requires thousands of data points to be highly accurate, although we overcame this limitation by constructing a thin-layer CNN model. Second, our regression CNN model underestimated PAWP in several patients with an extremely high GT PAWP. For example, in case 4 (see Fig. 4), the GT PAWP was 44.0 mm Hg but the estimated PAWP was 24.0 mm Hg. This poor estimate may be because the median GT PAWP in our dataset was 11 mm Hg (IQR 8–15 mm Hg) and few training data had a GT PAWP greater than or equal to 30 mm Hg. Third, we collected data retrospectively, and RHC and chest radiography were not performed simultaneously. Last, we excluded patients with lung diseases because we believed that lung lesions, such as pleural effusion or inflammation, could affect the learning process of deep learning. In addition, patients with percutaneous cardiopulmonary support and a left ventricular assist device were also excluded because the difficulty of performing RHC means that PAWP obtained in these patients may not be accurate and because these large medical devices may affect the learning process of deep learning.

In the future, this deep learning model should be improved by applying it in a larger cohort from a real-world population. Nevertheless, despite the above limitations, our results provide insight into the application of artificial intelligence and deep learning to cardiology.

In conclusion, regression CNN can be applied to quantitatively estimate PAWP from standard chest radiographs in patients with cardiovascular diseases.

## Supplementary Information

Below is the link to the electronic supplementary material.

Supplementary Fig. 1 Learning curves of the models by 5-hold cross-validation. The learning rates 10-5.5 (A), 10-6.0 (B), and 10-6.5 (C) are the mean squared errors (MSEs) of the main training data, and the learning rates 10-5.5 (D), 10-6.0 (E), and 10-6.5 (F) are the MSEs of the validation data (the construction of cross-validation datasets is shown in Figure 2). When the 2 higher learning rates were used (10-5.5 or 10-6.0), the trend of the MSE of the validation data was unstable. In contrast, when the lowest learning rate was used (10-6.5), the trend was stable and the MSE of the validation data achieved the minimum required value. cv id, cross-validation identification

Supplementary Fig. 2 Distribution of pulmonary artery wedge pressure in the training and test groups. PAWP, pulmonary artery wedge pressure
