# Supplementary figures and images for: Quantitative estimation of pulmonary artery wedge pressure from chest radiographs by a regression convolutional neural network
Source: Heart Vessels. 2022 Feb 27;37(8):1387–94. doi: 10.1007/s00380-022-02043-w (PMC9239946; doi:10.1007/s00380-022-02043-w)

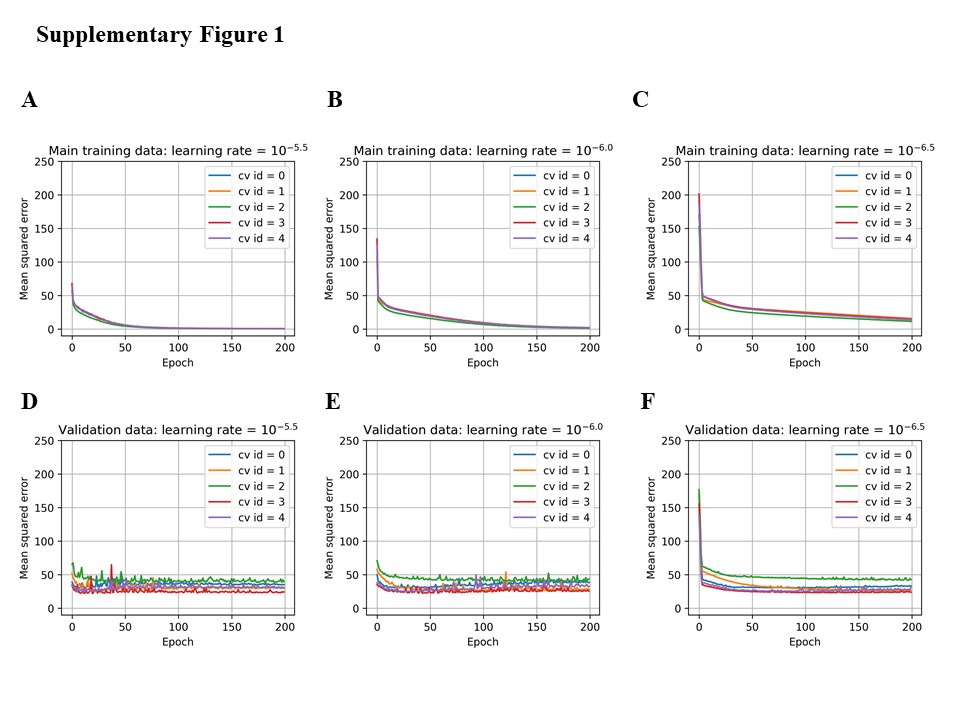

Supplement: Supplementary file 1 — Supplementary Fig. 1 Learning curves of the models by 5-hold cross-validation. The learning rates 10-5.5 (A), 10-6.0 (B), and 10-6.5 (C) are the mean squared errors (MSEs) of the main training data, and the learning rates 10-5.5 (D), 10-6.0 (E), and 10-6.5 (F) are the MSEs of the validation data (the construction of cross-validation datasets is shown in Figure 2). When the 2 higher learning rates were used (10-5.5 or 10-6.0), the trend of the MSE of the validation data was unstable. In contrast, when the lowest learning rate was used (10-6.5), the trend was stable and the MSE of the validation data achieved the minimum required value. cv id, cross-validation identification [file 380_2022_2043_MOESM1_ESM.jpg]

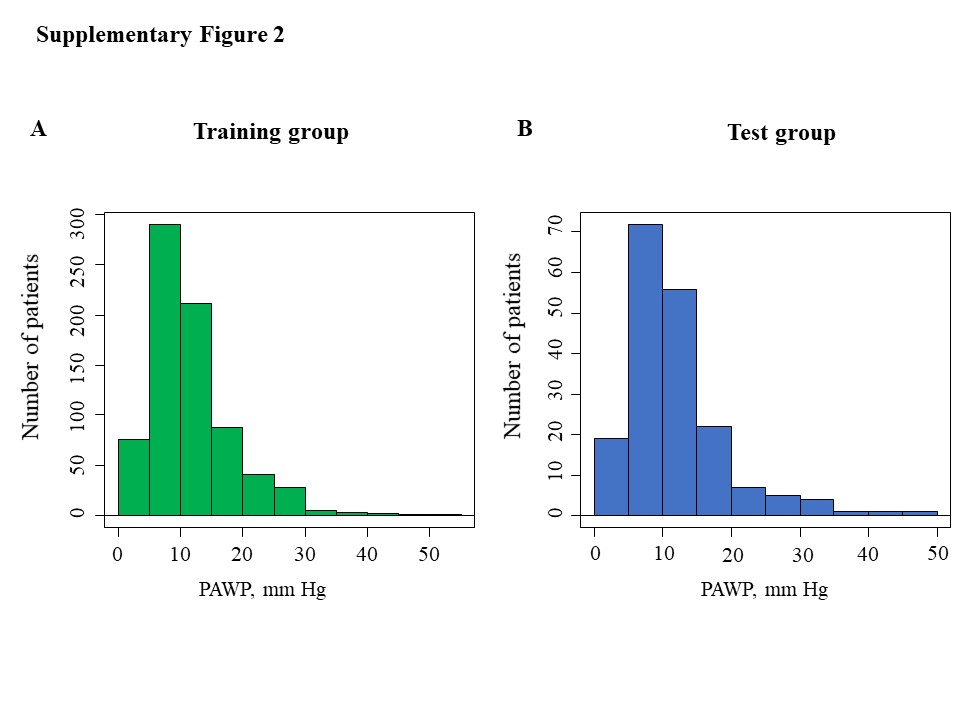

Supplement: Supplementary file 2 — Supplementary Fig. 2 Distribution of pulmonary artery wedge pressure in the training and test groups. PAWP, pulmonary artery wedge pressure [file 380_2022_2043_MOESM2_ESM.jpg]
